# First use of Braxon® acellular dermal matrix for complex revision aesthetic breast surgery—revision augmentation mastopexy

**DOI:** 10.1093/jscr/rjab256

**Published:** 2021-06-29

**Authors:** Christine Bojanic, Stavros Samaras, Mwenya M Chishimba, Charles M Malata

**Affiliations:** Department of Plastic & Reconstructive Surgery, Addenbrooke’s Hospital, Cambridge University Hospitals NHS Foundation Trust, Cambridge, UK; Department of Plastic & Reconstructive Surgery, Addenbrooke’s Hospital, Cambridge University Hospitals NHS Foundation Trust, Cambridge, UK; Department of Plastic & Reconstructive Surgery, 401 Military Hospital of Athens, Athens, Greece; School of Medicine & Dentistry, University of Alberta, Edmonton, Canada; Department of Plastic & Reconstructive Surgery, Addenbrooke’s Hospital, Cambridge University Hospitals NHS Foundation Trust, Cambridge, UK; Cambridge Breast Unit, Department of Surgery, Addenbrooke’s Hospital, Cambridge University Hospitals NHS Foundation Trust, Cambridge, UK; School of Medicine, Anglia Ruskin University, Cambridge, UK

**Keywords:** Braxon®, augmentation-mastopexy, revision implant surgery, implant malposition, acellular dermal matrix (ADM), capsular contracture

## Abstract

Acellular dermal matrices (ADMs) have ushered in a paradigm shift in prosthetic breast reconstruction; however, there has hitherto been no reported use of Braxon® ADM in aesthetic breast surgery. Here, we describe the case of a 42-year-old woman who presented for revision of her bilateral aesthetic augmentation-mastopexy following multiple revision surgeries. The predominant concerns were persistent pain, implant malposition and a wide intermammary distance. Her predicament was worsened by inability to tolerate monopolar diathermy owing to a spinal stimulator—the least invasive operation was sought and Braxon® ADM met this criterion. The procedure was a success, and she remains symptom-free, with soft breasts and stable implant positions. Braxon® ADM, with its preformed shape, total implant-wrapping design and easy suture fixation, lends itself to easy use in cosmetic breast surgery. Its role in cosmetic breast surgery has yet to be established, but this case marks the beginning of this endeavor.

## INTRODUCTION

Acellular dermal matrices (ADMs) were first described for reconstructive breast surgery in the early 2000’s [1] and are of porcine, bovine or human origin. Braxon® (Decomed SrL, Venezia, Italy), a pre-shaped bovine ADM, has created a paradigm shift in current prosthetic breast reconstruction practice—by the *ex-vivo* complete wrapping of an implant with an ADM prior to placement inside a prepectoral pocket facilitated by minimal fixation sutures and practically isolating the prosthesis from direct contact with the surrounding tissues [2–4]. ADMs have also been applied to aesthetic breast surgery for a variety of indications; however, there has been no reported case of Braxon® ADM use in revision aesthetic breast surgery [5–15]. These have included synmastia correction, inframammary fold definition, camouflage of rippling and upper pole irregularities, providing implant support and reinforcing lower pole breast tissue to prevent ‘bottoming out’ and providing an interface during capsulotomies [7, 10].

We present the first reported use of Braxon® for complex aesthetic revision surgery of breast asymmetry (with a tuberous component) as an adjunct to improve suboptimal aesthetics following staged augmentation-mastopexy complicated (at different times) by severe capsular contracture, implant rupture and significant implant malpositioning. The potential applications of total implant wrapping ADM in cosmetic breast surgery are also discussed.

## CASE REPORT

**Figure 1:**
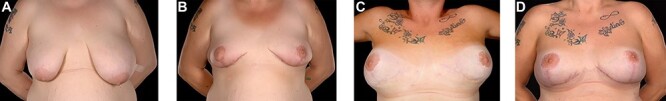
39-year-old with a history of severe breast asymmetry and mild tuberous breast deformity (**A**) corrected in two stages (left breast reduction and right mastopexy (**B**) followed by bilateral subpectoral augmentation 1 year later). This was complicated by left implant rupture and bilateral Baker III capsular contracture for which she underwent bilateral capsulectomies and implant exchange. The patient subsequently developed hypermobile implants with lateral displacement, a wide intermammary distance, and no cleavage (**C**) requiring Wise pattern mastopexy and exchange for smaller 370 and 325 cc (as former 590 and 525 cc could not be totally covered by the Braxon® ADM) implants with total Braxon® ADM coverage (**D**) which was secured to the chest wall with 2/0 PDS. Prior to Braxon®, a very wide intermammary distance (and loss of IMF definition) (**C**) due to stretched tissues following massive weight loss and combined with very large implants (subglandular implant position) can be seen.

**Figure 2:**
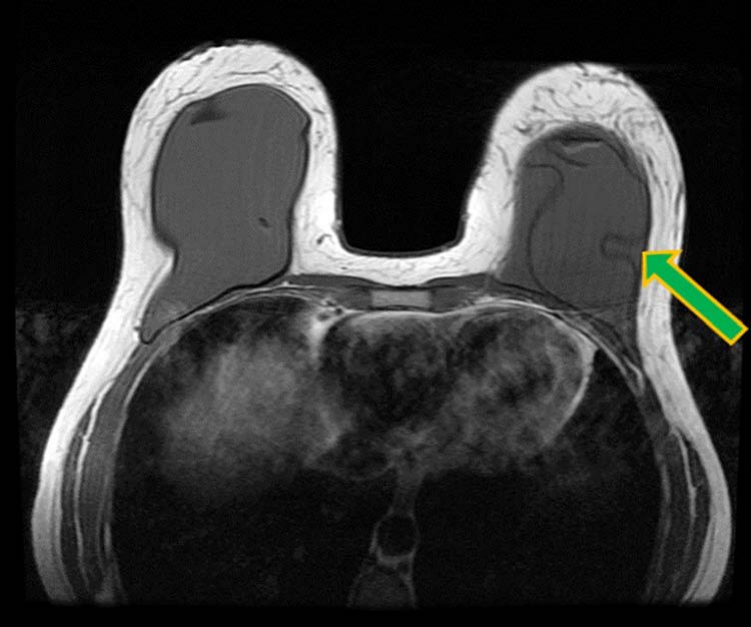
Ruptured left breast implant on MRI as demonstrated by the linguine sign (indicated by green arrow).

A 42-year-old woman presented for revision of her aesthetic bilateral breast augmentation-mastopexy following multiple implant-related complications. She had initially undergone correction of severe breast asymmetry with mild tuberous breast deformity and Grade-III ptosis in two stages 10 years earlier. Firstly, the breast asymmetry was corrected with a right mastopexy and left breast reduction (Fig. 1A). This was complicated by periareolar fat necrosis on the left and T-junction wound breakdown of the right breast which required debridement and split skin grafting. Subsequently, the second stage was performed comprising differential (inframammary subglandular) breast augmentation with round textured cohesive gel implants (McGhan style 110; left 450 g right 510 g, used prior to the 2018 EU-wide ban). Eight years later however, she represented with significant mastalgia and Baker III capsular contractures bilaterally (Fig. 1B). Having voluntarily lost 50 kg in weight, the patient also noted more prominent ripples in both breasts and an MRI was performed which confirmed a left breast implant rupture (Fig. 2). Bilateral total capsulectomies with implant exchange to anatomical texture silicone implants (Sebbin SM size 590 cc on the right and 525 cc on the left) were performed resulting in satisfactory shape, volume and projection (Fig. 1C). However, she returned 11 months later, complaining of pain associated with her implants and with the position of the implants especially as they dislodged laterally on her lying down (Fig. 1D). On examination, she had good symmetry, soft breasts; however, the implants were hypermobile with a wide intermammary distance and no cleavage (Fig. 1D). Her BMI was 26.4 kg/m^2^ (after weight loss), her bra cup size a 32F and she was a current smoker (~10 cigarettes per day for 13 years).

Owing to her having a spinal cord stimulator implant from a previous spinal operation, monopolar diathermy cautery during surgery was contra-indicated. The patient expressed that she did not want the implants to be removed since that would mean leaving her with smaller breasts. The use of Braxon® ADM for total coverage and anchoring of the implant to the chest wall was agreed and she accepted a smaller implant size (590 and 525 cc) to enable the Braxon® ADMs to be wrapped entirely.

**Figure 3:**
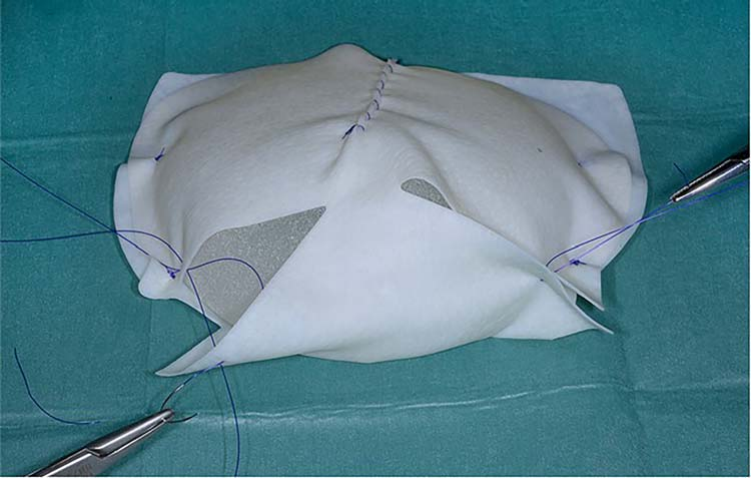
Braxon®-ADM totally wrapped around a fixed volume implant prior to insertion into the implant pocket.

Intraoperatively (under LA and only bipolar diathermy), a standard Wise-pattern mastopexy (superomedial pedicle) was performed with removal of both implants. The Braxon® ADM (30 × 20cm) was trimmed and wrapped around fixed volume silicone gel implants (Sebbin 325 ml (LSA-SL325) on the right and 370 ml (LSA-SL370) on the left) and secured with 3/0 polydioxanone suture (Fig. 3). The implant with its ADM covering was rinsed in 10% povidone iodine and then inserted (in existing subglandular pockets) and sutured to the chest wall with 2/0 polydioxanone suture medially, superiorly and laterally.

There were no peri-operative or post-operative complications, and she was discharged 2 days after the surgery after drain removal. The patient was seen in clinic 6 months later with a great cosmetic result, now wearing a 36DD bra, very happy with the size and shape of both breasts. Three years after her revision, she remains symptom-free, good position of the implants and no capsular contracture (Fig. 1D).

## DISCUSSION

Revisional aesthetic breast surgery can be quite challenging. The commonest implant-related complications, which require surgical intervention include among others implant malposition, capsular contracture, visible rippling, symmastia and ‘bottoming out’ [6]. ADMs have revolutionized primary and secondary breast reconstruction and have also begun to get a foothold in aesthetic breast surgery revisions [1, 5–15].

To date, ADMs have been successfully used in aesthetic breast surgery for revision of mastopexy, correction of symmastia, improvement of inframammary fold definition and ‘bottoming out’, treatment of implant malposition, correction of implant rippling, provision of an interface when performing capsulectomies, and as an adjunct to the overlying thinned parenchyma to reinforce the implant cover and mask the associated wrinkling [5–15].

Braxon®, with its preformed shape, total implant wrapping and easy internal suture fixation, lends itself easy to use in cosmetic breast surgery. However, to our knowledge (after an extensive literature search), it has hitherto not been applied in this fashion.

The Braxon® ADM was used to reinforce the weak stretched tissues and to ensure that the implant was kept in position, thus effectively treating the implant malposition and hyper-mobility. Braxon® total additional cover and fixation was particularly useful as the implants were in the subglandular position where implant mobility can be a problem with poor quality breast tissues. Lastly, Braxon® supported the attenuated IMF and the lower pole tissues, thus preventing implant ‘bottoming out’.

The first use of pre-shaped Braxon® ADM with total implant coverage in a revision augmentation-mastopexy is hereby presented. Its ease of use opens its potential cosmetic indications in revision cosmetic breast surgery ranging from mastopexy, augmentation and combined augmentation-mastopexy.

## CONFLICT OF INTEREST STATEMENT

The authors have no financial or other interest in Braxon® and are not in any way linked to Raise Healthcare, the UK distributors of Braxon® or Decomed SrL (Italy), the manufacturers of Braxon®. The senior author (C.M.M.) had his travel expenses reimbursed for lecturing at two Braxon® symposia organized by Raise Healthcare UK for no remuneration.
